# Burnout, grit and resilience among Jordanian orthopedic surgeons: a cross-sectional study

**DOI:** 10.1186/s12909-023-04572-y

**Published:** 2023-08-21

**Authors:** Mohammad Hamdan, Bassem I. Haddad, Mohammad Ali Alshrouf, Abdallah Al-Ani, Mohammed S Alisi, Yazan Hammad, Ahmad Alelaumi, Hashem Al Hawamdeh, Ahmad Abu Halaweh, Lara Alshabatat, Sanad Kawasmi

**Affiliations:** 1 Department of Special Surgery, Division of Orthopedics, School of Medicine, The University of Jordan, Amman, 11942 Jordan; 2 Jordan University Hospital, The University of Jordan, 11942 Amman, Jordan; 3 Office of Scientific Affairs and Research, King Hussein Cancer Center, Amman, Jordan; 4 Faculty of Medicine, Islamic University of Gaza, Gaza, Palestine; 5 Department of Orthopedics and Spine Surgery, King Hussein Cancer Center, Amman, Jordan; 6 School of Medicine, The University of Jordan, Amman, 11942 Jordan

**Keywords:** Burnout, Grit, Resilience, Jordan, Orthopedics, Surgeons

## Abstract

**Background:**

Burnout is a serious issue that affects physicians more than the general population; however, those with higher levels of grit and resilience have been shown to experience lower levels of burnout. The primary aim was to determine the prevalence of burnout among Jordanian orthopedic surgeons, explore its risk factors, and investigate the relationship between burnout and grit and resilience.

**Methods:**

We conducted a questionnaire-based cross-sectional study targeting a total of 180 orthopedic surgeons attending the yearly Jordanian National Orthopedic Conference (JNOC). Non-random sampling (i.e., convenience) was utilized to recruit participants. The abbreviated Maslach Burnout Inventory, short grit scale, and Connor-Davidson Resilience Scale were used. Scores were examined using the Mann–Whitney U, Kruskal–Wallis H, and Spearman’s rho tests, of which results were corrected using the Bonferroni method.

**Results:**

Among 135 respondents, 62.2% were specialists and 37.8% were residents. About 52.0% practiced in public hospitals. Approximately 69.0% worked for more than 50 h weekly. The prevalence of burnout among all participants was 45.2% with more frequency among residents (66.7%). Burnout and the participants’ grit and resilience showed an inverse relationship (ρ = -0.441 and ρ = -0.312, respectively). Age (ρ = 0.337), number of children (ρ = 0.245), and years of experience (ρ = 0.331) were positively correlated with grit. The median score for grit was higher in physicians who had or are having their residency outside Jordan (p < 0.001). Age (ρ = 0.233) and years of experience (ρ = 0.269) were positively correlated with resilience.

**Conclusion:**

Jordanian orthopedic surgeons face significant rates of burnout. Institutional efforts should be taken to detect and prevent burnout in addition to enhancing the grit and resilience among orthopedic professionals.

## Introduction

The body of literature is tremendously growing regarding burnout among physicians, including their different stages of study and training [1]. Many definitions have been suggested for burnout [2]. Maslach and Jackson in 1981 defined it as the consequence of emotional exhaustion, depersonalization, and a reduced sense of accomplishment in the healthcare field, and to make it easier for studying and comparing, they categorized it into three main components, including emotional exhaustion, depersonalization, and a reduced sense of personal accomplishment [3]. Increased emotional exhaustion and depersonalization scores indicate excessive burnout, while a high sense of personal accomplishment score indicates otherwise.

Several studies raise the issue that burnout among physicians can reflect on different aspects of their physical, psychological, and social lives, with suicide arguably being the one of most serious concerns [2, 4, 5]. Suicide was reported as the leading and second cause of death among male and female U.S. resident physicians, respectively [6]. Recently, it was reported that the suicide rate is highest among orthopedic surgeons in particular [7]. In addition, the repercussions of burnout can be very dangerous for patients, who are more likely to be exposed to medical errors when treated by burned-out or less satisfied clinicians [8, 9]. Moreover, burnout has been said to be associated with unsatisfactory patient treatment, decreased patient satisfaction, lower access to care, and higher health-care expenditures [10]. As a result, burnout can have an adverse effect on the quality of care provided, rather than just the physician.

It has been reported that burnout is higher in orthopedic surgeons than in general surgeons, reaching up to 60% [11]. According to a systematic review of healthcare professionals in the Middle East, Chemali et al., found that between 40 and 60% of these professionals were experiencing burnout [12]. As burnout becomes a more obvious issue, it is necessary to counteract its effects. Shakir et al., reported an inverse relationship between grit and resilience and the presence of burnout among neurosurgery residents [13]. Grit is commonly measured using the short grit scale by focusing on the individual’s persistence and long-term commitment [14]. Grit was found to be important for completing surgical training [15]. A resilient physician is defined as the one who can bounce back after difficulties while also being stronger [16]. Lack of resilience can result in increased stress and ill health for medical staff [17].

Within the Middle East region, reports examining the dynamics between grit, resilience, and burnout are scant. Al-Zain and Abdulsalam studied Saudi Arabian dental students and revealed that burnout was negatively correlated with both resilience and grit [18]. Similarly, Alsharif et al., demonstrated that burnout was negatively correlated with resilience among Dental students in Western Saudi Arabia [19]. Additionally, Al-Qahtani et al., found that among Saudi university students, grit is inversely related to neurological fatigue, cognitive failure and apathy but positively correlated with anxiety and depression [20]. Finally, a collaborative study involving pharmacy students within 14 Asian and Middle Eastern countries had revealed that higher grit is associated with exercise, academic performance, and type of university among others [21]. However, the study did not investigate the dynamics between grit and resilience or burnout, which is similar to other Middle Eastern reports concerning with grit and academic performance [22, 23].

Burnout among physicians was deeply studied over the last decade. However, research on burnout with regard to grit and resilience has not been thoroughly addressed, especially in our region. The aim of this study was to investigate the relationship between grit and resilience to burnout among orthopedic surgeons in Jordan. In addition, to determine the factors associated with burnout, grit, and resilience. We hypothesized that with increased grit and resilience, there would be a decrease in burnout.

## Materials and methods

### Study design and population

This study utilized a cross-sectional observational design that took place during the yearly Jordanian National Orthopedic Conference (JNOC). It involved an anonymous questionnaire distributed to the attendees during the conference. The conference had around 180 orthopedic residents and specialists from different sectors and regions of the country. The study included all orthopedic residents and attending physicians/specialists who gave voluntary consent and completed the survey. All participants were recruited through non-random sampling (i.e., convenience sampling). Ethical approval was granted by the Institutional Review Board (IRB) of the Jordan University Hospital (reference number: 1,020,208,578).

Sample size calculation was conducted on G*power (ver. 3.1.9.7). Using the following parameters (effect size, 0.5; alpha error, 0.05; power; 0.80), a minimum sample of 126 participants was needed to conduct statistical tests for mean differences of appropriate rigor.

### Study tool

We have developed an anonymous self-administered questionnaire to measure the study’s endpoints. The questionnaire contained 4 sections: first, the demographics section (age, gender, marital status, number of children, place where children live, country where orthopedics training was done or is being done, residency year or experience year, city of practice, work sector, subspecialty in orthopedics, country of graduation from medical school, and weekly working hours), and a 5-point scale question on social and personal stressors outside of work, ranging from 0 (not at all) to 4 (extreme), which was adapted from a previous study [13].

The second section contained 9-items that represent the abbreviated Maslach Burnout Inventory (aMBI), derived from the 22-item Maslach Burnout Inventory-Human Services Scale, to assess the three components of burnout, including personal accomplishment (PA), emotional exhaustion (EE), and depersonalization (DP) subscales [3, 24]. Each component had 3-items and was scored on an 18-point scale and categorized as low (0–6 points), intermediate (7–12 points), or high (13–18 points). A high score of either EE or DP indicated the presence of burnout.

The third section was the short grit scale (SGS), which contained 8-items to assess the grit of the participant [14]. Each item is scored from 1 to 5. The sum of all items is then divided by 8 to produce a final score between 1 (not gritty at all) to 5 (extremely gritty). The fourth section assessed resilience using the Connor-Davidson Resilience Scale (CD-RISC 10), which is a 10-item survey rated on a 5-point scale, ranging from 0 (not true at all) to 4 (true nearly all the time), with higher scores reflecting greater resilience [25].

The face and content validity of the questionnaire were reviewed before starting data collection by a panel of consultant orthopedic surgeons and questioner experts to make sure that the questions were clear and covered the data that were required to assess various aspects of the study. Reliability was examined by calculating Cronbach’s alpha for the aMBI, SGS, and CD-RISC 10, and the values were found to be 0.676, 0.633, and 0.944, respectively.

### Statistical analysis

Statistical analysis was performed using SPSS version 29.0 (Chicago, IL, USA). The mean ± standard deviation or medians and interquartile ranges (IQRs) described continuous variables (e.g., age) and count (frequency) were used to describe the nominal variables (e.g., gender). Shapiro-wilk test was used to check the normality. The relationships between aMBI, SGS, and CD-RISC 10 and participant characteristic was assessed using Mann–Whitney U test or Kruskal–Wallis H test. All results were corrected using the Bonferroni correction (significant p value < 0.005). The Spearman’s rank correlation (ρ) was used to calculate the correlation between continuous variables. Results of the correlations analysis were corrected for multiple comparisons using the Bonferroni correction (significant p value < 0.0002). Residents were categorized into junior orthopedics residents (first, second, and third years) and senior residents (fourth and fifth years). Similarly, specialists were categorized per years of experience (> 10 years vs. <10 years). A p-value of < 0.05 was considered statistically significant.

## Results

### Characteristics of the study population

In total, 135 physicians completed the questionnaire and were included in the analysis, which yields a response rate of 75%. The mean age of the participants was 39.10 ± 11.26 years, ranging from 24 to 72. Eighty-four (62.2%) were specialists and 51 (37.8%) were residents. The majority (66.6%, n = 34) of residents were fourth- and fifth-year residents. The majority of respondents were orthopedic trauma surgeons (29 out of 85, 34.1%). More than two thirds of the participants (73.3%) were married (89.3% of specialists compared to 47.1% of residents). Only three participants were separated/divorced/widowed; all of them were specialists. Married participants had a mean of 2.72 ± 1.53 children (range, 0–8).

More than half (51.9%) graduated from local universities with MBBS or MD degrees. About two thirds of specialists (69.0%) completed their orthopedic residency training in Jordan, and the majority (94.1%) of residents are completing their residency in Jordan. Nearly half (51.9%) of the participants were practicing in public hospitals, 28.9% in the private sector, and 19.3% in university hospitals. Of the 135 participants, 61.5% were working in the capital city and the others were distributed over other cities.

Regarding social and personal stressors outside work, most of the participants (69.6%) reported mild or moderate stress outside work. In addition, more than two thirds of the participants (68.8%) worked for more than 50 h weekly. Table 1 illustrates the sociodemographic characteristics of the participants.

**Table 1:** Demographic characteristics of the participants

Characteristic		n (%) or mean ± SD
Age		39.10 ± 11.26
Marital Status	Married	99 (73.3)
	Single	33 (24.4)
	Separated/divorced/widowed	3 (2.2)
Number of children		2.72 ± 1.53
Place where children live	Living in the same home	84 (88.4)
	Living in another home	11 (11.6)
City of practice	Amman	83 (61.5)
	Other	52 (38.5)
Position	Resident	51 (37.8)
	Specialist	84 (62.2)
Residency year	First	4 (7.8)
	Second	6 (11.8)
	Third	7 (13.7)
	Fourth	22 (43.1)
	Fifth	12 (23.5)
Specialty	Trauma	29 (34.1)
	Arthroplasty	19 (22.4)
	Pediatric	13 (15.3)
	Upper Limb	11 (12.9)
	Sport	7 (8.2)
	Foot	2 (2.4)
	Hand	2 (2.4)
	Spine	2 (2.4)
Specialists’ years of experience		14.26 ± 9.56
Work Sector	Public	70 (51.9)
	Private	39 (28.9)
	University hospital	26 (19.3)
Medical school	Jordanian medical school	70 (51.9)
	Foreign medical school	65 (48.1)
Orthopedics residency training	Jordan	106 (78.5)
	Outside	29 (21.5)
Social/ personal stressor outside work	Not at all	28 (20.7)
	Small amount	50 (37)
	Moderate amount	44 (32.6)
	Large amount	9 (6.7)
	Extreme	4 (3)
Weekly working hours	31–40	13 (9.6)
	41–50	29 (21.5)
	51–60	40 (29.6)
	61–70	20 (14.8)
	≥ 71	33 (24.4)

Overall, the prevalence of burnout among all participants was 45.2% with more frequency in residents (66.7%) than in specialists (32.1%). Table 2 illustrates the rate of the three main components of burnout scale (EE, DP, and PA) for all recruited participants.

**Table 2:** Illustrates the prevalence of burnout and level of its 3 components among residents and specialists

MBI-HSS Components		Resident	Specialist/Consultant	Total
Lack of personal accomplishment	Low	1 (2.0%)	0 (0.0%)	1 (0.7%)
	Intermediate	10 (19.6%)	9 (10.7%)	19 (14.1%)
	High	40 (78.4%)	75 (89.3%)	115 (85.2%)
Emotional Exhaustion	Low	8 (15.7%)	29 (34.5%)	37 (27.4%)
	Intermediate	10 (19.6%)	28 (33.3%)	38 (28.1%)
	High	33 (64.7%)	27 (32.1%)	60 (44.4%)
Depersonalization	Low	12 (23.5%)	65 (77.4%)	77 (57.0%)
	Intermediate	24 (47.1%)	16 (19.0%)	40 (29.6%)
	High	15 (29.4%)	3 (3.6%)	18 (13.3%)
Burnout	No	17 (33.3%)	57 (67.9%)	74 (54.8%)
	Burnout	34 (66.7%)	27 (32.1%)	61 (45.2%)

### Factors affecting burnout

**Fig. 1:**
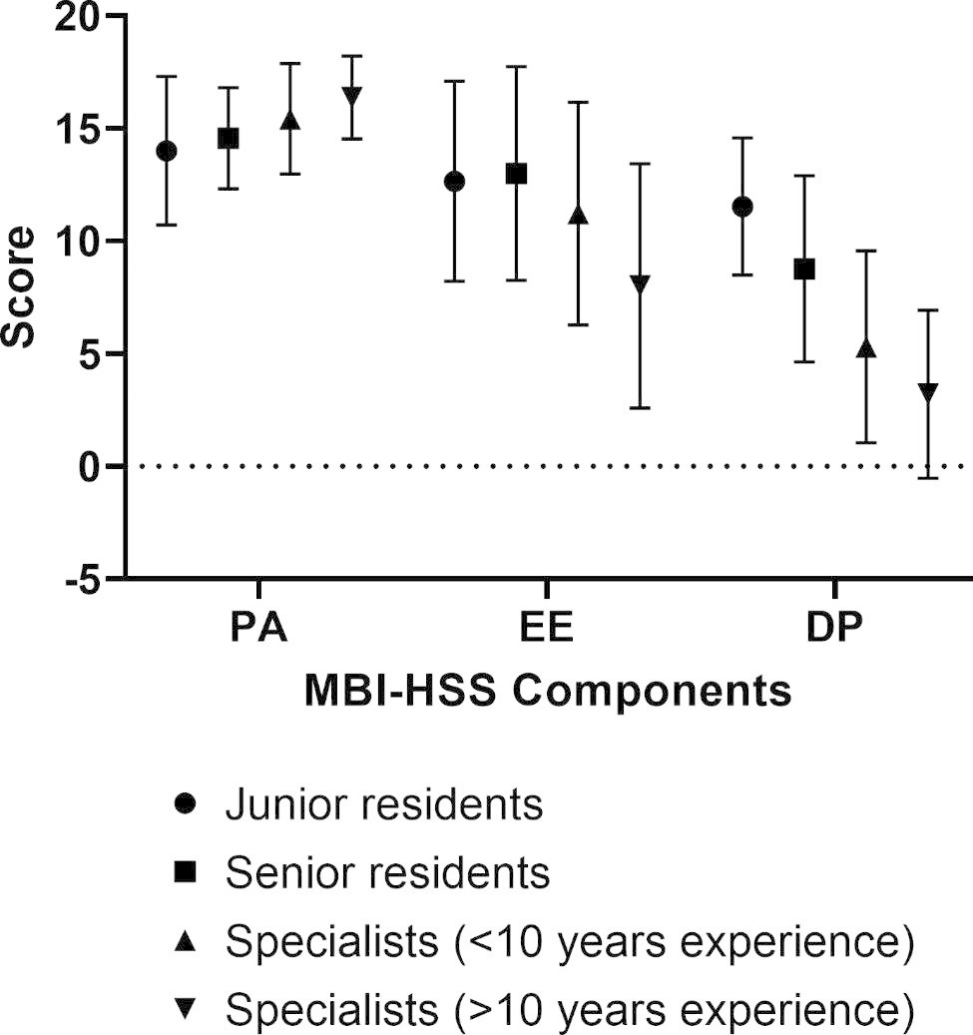
Box plots diagram showing comparative details of median, quartile and extreme values of personal accomplishment (PA), emotional exhaustion (EE), and depersonalization (DP) subscales

Age was positively correlated with PA (ρ = 0.348, p < 0.001) and negatively with EE (ρ = -0.408, p < 0.001) and DP (ρ = -0.601, p < 0.001). Number of children was only negatively correlated with DP (ρ = -0.377, p < 0.001). Years of experience among specialists were negatively correlated with EE (ρ = -0.380, p < 0.001) and DP (ρ = -0.435, p < 0.001). The median score for PA was higher among specialists (p < 0.001), particularly those with more than 10 years of experience. The median score for EE was higher in residents (p < 0.001), physicians who worked in the public sector (p < 0.001), physicians who had or is having their residency in Jordan (p < 0.001), physicians who had high social or personal stressors outside work (p = 0.002), and long working hours (p < 0.001). The median score for DP was higher in those who were single (p < 0.001), residents (p < 0.001), physicians who had or are having their residency in Jordan (p < 0.001), and long working hours (p < 0.001). Table 3; Fig. 1 demonstrate the relationships between the participants characteristic and burnout scale (EE, DP, PA).

**Table 3:** Abbreviated Maslach Burnout Inventory components among the participants

Characteristic		Median (IQR) or Spearman’s ρ					
		PA score	p-value	EE score	p-value	DP score	p-value
Age		0.348	< 0.001^a^	-0.408	< 0.001^a^	-0.601	< 0.001^a^
Marital Status	Married	16 (4)	0.032^b^	10 (10)	0.018^b^	4 (6)	< 0.001^b^
	Single	15 (3)		14 (5)		9 (7)	
	Separated/divorced/widowed	17 (2)		5 (14)		3 (3)	
Number of children		0.184	0.184^a^	-0.272	0.007^a^	-0.377	< 0.001^a^
Place where children live	Living in the same home	16 (4)	0.101^c^	10 (8.5)	0.031^c^	4 (6)	0.063^c^
	Living in another home	17 (3)		6 (8)		1 (5)	
City of practice	Amman	16 (3)	0.708^c^	13 (8)	0.018^c^	6 (8)	0.106^c^
	Other	16 (4)		10 (10.5)		4.5 (8)	
Position	Resident	15 (3)	< 0.001^c^	14 (7)	< 0.001^c^	9 (5)	< 0.001^c^
	Specialist	17 (3)		9 (9)		3 (5)	
Residency year	First	14 (7)	0.828^b^	9 (11.5)	0.761^b^	11.5 (4.5)	0.020^b^
	Second	15 (2)		14.5 (3)		14.5 (3)	
	Third	14 (5)		13 (7)		9 (1)	
	Fourth	15.5 (3)		14.5 (7)		8 (6)	
	Fifth	14.5 (3)		15 (8)		8.5 (5)	
Specialty	Trauma	17 (4.5)	0.710^b^	8 (11)	0.654^b^	4 (7)	0.172^b^
	Arthroplasty	16 (5)		11 (10)		4 (4)	
	Pediatric	15 (3)		10 (6)		4 (5)	
	Upper Limb	15 (3)		8 (11)		8 (8)	
	Sport	16 (2)		11 (8)		9 (10)	
	Other	15.5 (3)		8 (10)		1.5 (2)	
Specialists’ years of experience		0.146	0.185^a^	-0.380	< 0.001^a^	-0.435	< 0.001^a^
Work sector	Public	16 (3)	0.872^b^	13.5 (8)	< 0.001^b^	7 (8)	0.014^b^
	Private	16 (4)		8 (11)		3 (8)	
	University hospital	16 (3)		8.5 (9)		5 (7)	
Medical school	Jordanian medical school	16 (3)	0.046^c^	13 (7)	0.031^c^	6.5 (7)	0.018^c^
	Foreign medical school	16 (4)		9 (10)		4 (8)	
Orthopedics residency training	Jordan	16 (3)	0.209^c^	13 (7)	< 0.001^c^	6 (7)	< 0.001^c^
	Outside	16 (4)		6 (7)		1 (7)	
Social/ personal stressor outside work	Not at all	16.5 (3.5)	0.303^b^	10 (11)	0.002^b^	5 (7.5)	0.251^b^
	Small amount	16 (4)		9 (9)		4 (7)	
	Moderate amount	16 (4)		14.5 (7.5)		7 (8.5)	
	Large amount	16 (1)		10 (7)		7 (4)	
	Extreme	17 (4)		17 (3)		9 (10.5)	
Weekly working hours	31–40	16 (2)	0.713^b^	5 (4)	< 0.001^b^	4 (10)	< 0.001^b^
	41–50	17 (3)		9 (9)		3 (4)	
	51–60	16 (4)		11 (9)		5 (5.5)	
	61–70	15.5 (2.5)		10.5 (9)		7 (6.5)	
	≥ 71	16 (3)		15 (5)		9 (6)	

Note: ^a^ Spearman’s rank correlation, ^b^ Kruskal–Wallis H, ^c^ Mann–Whitney U test; Mann–Whitney U test and Kruskal–Wallis H test results were corrected using Bonferroni correction

### Factors affecting grit and resilience

Age (ρ = 0.337, p < 0.001), number of children (ρ = 0.245, p = 0.012), and years of experience among specialists (ρ = 0.245, p = 0.012). The median score for grit was higher in physicians who had or are having their residency outside Jordan (p < 0.001). Table 4 demonstrate the relationships between the participants’ characteristics and grit.

**Table 4:** Short grit scale among the participants

Characteristic		Median (IQR) or Spearman’s ρ	
Age		0.337	< 0.001^a^
Marital Status	Married	3.6 (1)	0.040 ^b^
	Single	3.4 (0.8)	
	Separated/divorced/widowed	3.6 (0.3)	
Number of children		0.245	0.012 ^a^
Place where children live	Living in the same home	3.6 (0.9)	0.007 ^c^
	Living in another home	4.1 (0.5)	
City of practice	Amman	3.5 (0.9)	0.157 ^c^
	Other	3.6 (1)	
Position	Resident	3.4 (0.8)	0.026 ^c^
	Specialist	3.6 (0.9)	
Residency year	First	3.1 (0.4)	0.022 ^b^
	Second	2.7 (0.5)	
	Third	3.3 (0.7)	
	Fourth	3.6 (1.1)	
	Fifth	3.7 (0.6)	
Specialty	Trauma	3.8 (1)	0.759 ^b^
	Arthroplasty	3.5 (0.8)	
	Pediatric	3.6 (0.4)	
	Upper Limb	3.3 (1.1)	
	Sport	3.5 (0.2)	
	Other	3.7 (0.6)	
Specialists’ years of experience		0.331	0.002 ^a^
Work Sector	Public	3.4 (0.7)	0.094 ^b^
	Private	3.8 (1.2)	
	University hospital	3.6 (1)	
Medical school	Jordanian medical school	3.4 (0.8)	0.309 ^c^
	Foreign medical school	3.5 (1)	
Orthopedics residency training	Jordan	3.5 (0.8)	< 0.001 ^c^
	Outside	4 (0.8)	
Social/ personal stressor outside work	Not at all	3.8 (1.2)	0.283 ^b^
	Small amount	3.6 (0.8)	
	Moderate amount	3.4 (0.8)	
	Large amount	3.5 (0.6)	
	Extreme	3.1 (1)	
Weekly working hours	31–40	3.5 (0.9)	0.749 ^b^
	41–50	3.6 (1)	
	51–60	3.5 (0.9)	
	61–70	3.8 (0.8)	
	≥ 71	3.4 (0.9)	

Note: ^a^ Spearman’s rank correlation, ^b^ Kruskal–Wallis H, ^c^ Mann–Whitney U test; Mann–Whitney U test and Kruskal–Wallis H test results were corrected using Bonferroni correction

Similarly, age and years of experience among specialists were positively correlated with resilience (ρ = 0.233, p < 0.001 and ρ = 0.269, p = 0.013, respectively). Table 5 demonstrate the relationships between the participants’ characteristics and resilience.

**Table 5:** Connor-Davidson resilience Scale among the participants

Characteristic		Median (IQR) or Spearman’s ρ	p-value
Age		0.233	0.007 ^a^
Marital Status	Married	31 (10)	0.211 ^b^
	Single	29 (10)	
	Separated/divorced/widowed	30 (19)	
Number of children		0.198	0.051 ^a^
Place where children live	Living in the same home	30 (9.5)	0.294 ^c^
	Living in another home	34 (10)	
City of practice	Amman	29 (12)	0.078 ^c^
	Other	30 (11)	
Position	Resident	29 (16)	0.038 ^c^
	Specialist	31 (10)	
Residency year	First	24.5 (16)	0.846 ^b^
	Second	27.5 (10)	
	Third	29 (22)	
	Fourth	28.5 (17)	
	Fifth	30 (7.5)	
Specialty	Trauma	31 (11)	0.587 ^b^
	Arthroplasty	29 (20)	
	Pediatric	32 (8)	
	Upper Limb	23 (16)	
	Sport	29 (7)	
	Other		
Specialists’ years of experience		0.269	0.013 ^a^
Work Sector	Public	28 (13)	0.0034 ^b^
	Private	29 (10)	
	University hospital	33.5 (5)	
Medical school	Jordanian medical school	30 (8)	0.583 ^c^
	Foreign medical school	29 (12)	
Orthopedics residency training	Jordan	29 (9)	0.007 ^c^
	Outside	34 (9)	
Social/ personal stressor outside work	Not at all	29 (15)	0.209 ^b^
	Small amount	31 (8)	
	Moderate amount	29 (11)	
	Large amount	29 (6)	
	Extreme	17 (23.5)	
Weekly working hours	31–40	29 (7)	0.425 ^b^
	41–50	31 (8)	
	51–60	29 (10)	
	61–70	31 (6.5)	
	≥ 71	28 (16)	

Note: a Spearman’s rank correlation, b Kruskal–Wallis H, c Mann–Whitney U test; Mann–Whitney U test and Kruskal–Wallis H test results were corrected using Bonferroni correction

### Correlations between PA, EE, DP, grit, and resilience

**Fig. 2:**
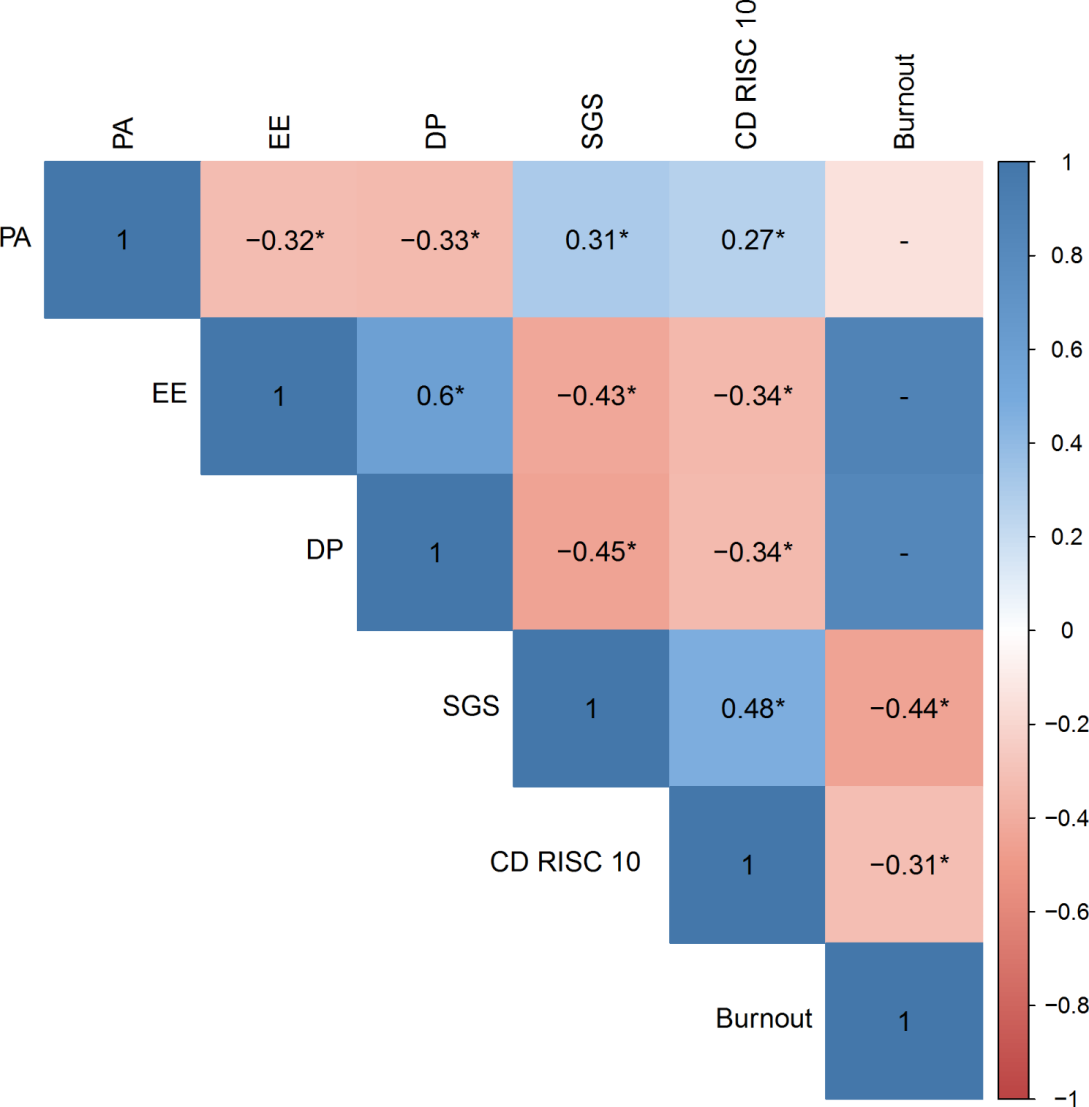
Correlation between personal accomplishment, emotional exhaustion, depersonalization, grit, and resilience; PA: personal accomplishment; EE: Emotional Exhaustion; DP: Depersonalization; SGS: short grit scale; CD-RISC 10: Connor-Davidson Resilience Scale; all correlation is significant at the 0.01 level (2-tailed)

The analysis of correlation between burnout and the participants’ grit and resilience showed an inverse relationship ρ = -0.441, p < 0.001 and ρ = -0.312, p < 0.001, respectively). PA and the participants’ grit and resilience demonstrated a positive relationship (ρ = 0.309, p < 0.001 and ρ = 0.267, p = 0.0017, respectively). A negative correlation was found between grit and resilience with EE (ρ = -0.427, p < 0.001 and ρ = -0.340, p < 0.001, respectively) and DP (ρ = -0.449, p < 0.001 and ρ = -0.336, p < 0.001, respectively). A significant positive correlation was found between participants’ grit and resilience (ρ = 0.477, p < 0.001) (Refer to Fig. 2).

## Discussion

We found that burnout was inversely correlated with participants’ grit and resilience. Moreover, the prevalence of burnout was higher among residents than among specialists. PA was negatively correlated with age and position. On the other hand, EE and DP were inversely correlated with age, years of experience, location of residency, as well as working hours. In addition, EE was higher in physicians who worked in the public sector and in physicians who had high social or personal stressors outside of work. DP was higher in physicians who had fewer children or were single. Moreover, grit was positively correlated with age, number of children, years of experience, and location of residency. Finally, resilience was only positively correlated with years of experience and age.

Burnout is a major concern for healthcare systems. In fact, orthopedic surgery is one of the surgical specialties that has significant burnout rates higher than those of general surgeons [26]. However, despite the high rates of burnout among orthopedic surgeons, little research has been done in this area. To the best of our knowledge, there exists no publications examining the rate of burnout among Jordanian orthopedic doctors nor the factors contributing to it. As of today, the Jordanian literature has only three articles generally examining stress and burnout among Jordanian physicians, of which some are associated with questionable methodological quality [27–29].

On a global scale, burnout in orthopedic surgeons ranged from 40 to 60%, with a higher prevalence among residents and department chairpersons [11, 26, 30]. In a systematic review investigating burnout among physicians in general, Roteinstein et al., reported a burnout prevalence of 67.0% [2]. A recent report documents that the prevalence of burnout within the Middle East ranges from 40 to 60% [12]. Interestingly, the aforementioned Jordanian reports found that 77.5% of the residents suffer from work-related burnout [27]. It was also demonstrated that 53.6% of residents had a high grade of emotional exhaustion [29]. Moreover, the majority of Jordanian residents felt nervous and stressed, with 73% having moderate levels of stress, and 18% having high levels of stress [28]. Our results showed a lower rate of burnout, reaching 45.2%, with more frequency among residents (66.7%) than specialists (32.1%).

Several factors have been linked to an increased rate of burnout in orthopedic surgeons. These include the nature of the specialty (i.e., physically demanding), workload, the working environment, time spent with family and close friends, and home life satisfaction [7, 31]. EE and DP were negatively correlated with years of experience among specialists, which was similar to previous studies which found that more years in practice were protective against burnout [31]. Our results revealed that residents had lower EE and DP scores, and the burnout rate was 66.7% among residents, which could be explained by the long working hours reported in our study.

In Jordan, it was reported by Al-Taher et al., that 82.4% of Jordanian residents had exceeded the 24-hour shift length threshold. In a previous study, they found those who worked more than 80 h had higher rates of burnout (69.2%), compared to 38.5% among those who worked fewer hours [32]. Moreover, a Jordanian report documented that residents of private and university hospitals had lower burnout levels [27]. In a recent study in Jordan, work-life balance was reported to be highly prevalent, with 62.9% of practicing physicians in Jordan reporting having work-life conflict [33]. Similarly, we have demonstrated that orthopedic residents in private and university hospitals had lower levels of EE. Additionally, our study showed that working long hours per week was significantly associated with higher EE and DP scores. According to a survey of internal medicine residents conducted by Gopal et al., reducing physicians’ work hours has been linked to lower rates of EE, DP, and depression but may also be related to education and quality of care [34]. While the presence of burnout may impact practitioners’ skills and medical knowledge, it may also precipitate significant losses in productivity [35].

Grit and resilience showed an inverse relationship with burnout in our study. Such phenomenon was previously documented in the literature as it has been found that those with higher levels of grit and resilience were less likely to experience burnout and poor health outcomes [13, 36, 37]. Burnout is a multidimensional and multifactorial issue [38]. It is influenced by a variety of factors including lack of social support, increased workload, and lack of mentorship to name a few. Moreover, the amount of burnout is modulated by individual level characteristics (e.g., age, personality traits) [38, 39]. This multifaced nature leads to almost a unique experience of burnout among residents. It appears that grit, a unique personality trait, possess protective effects against burnout up until a certain threshold [40, 41]. Evidence shows that levels of early grit during residency are able to predict burnout later during training [38]. The threshold seems to be impacted by other factors such as emotional intelligence, self-efficacy, and social belonging; all of which represent an individual’s ability to cope with difficult situations and are all associated with resilience [13]. In short, this consistent correlation between grit, resilience, and burnout showcases that individuals who can maintain their focus and commitment to goals (i.e., grit) and are able to adapt to and recover from adversity (i.e., resilience) are more likely to succeed within their respective fields.

A number participants’ characteristics were associated with grit, resilience and burnout. Our results showed that specialists who completed their residency training outside the country reported significantly higher grit scores. This might be explained by considering the effect that living abroad has on a person’s character and how the exposure to more challenges would build up an individual’s personality. In a multicenter cross-sectional study among UK doctors, they found a weak negative correlation between grit and burnout and a higher grit score among consultants compared to trainees [40]. Moreover, lower levels of grit were previously linked to a higher likelihood among residents to leave their training program [42]. Orthopedic surgery has the most competitive selection processes among all other programs throughout Jordan, thus, it is only expected for its applicant to have considerable scores for grit and resilience. Only the ones who are eager to learn, hard-working, committed, aware of the upcoming difficulties, and able to work under pressure are often chosen.

Several studies discussed feasible strategies to ameliorate the rates of burnout among healthcare workers. Implementing strategies and policies for prevention and identification of early signs of burnout were emphasized in more than one study [30, 43, 44]. At an institutional level, we would suggest that healthcare professionals, including orthopedic care providers, should be involved in programs focusing on providing trainees with coping mechanisms while also augmenting their grit and resilience. Moreover, institutions should subject their staff to regular checkups for early signs of burnout such as irritability, withdrawal, and family conflicts. Limiting the working hours, organizing the workload between the staff, mentorship, and professional appreciation are highly recommended [45].

Physician burnout has far-reaching effects, including on interactions with patients and colleagues. In order to allow participants to spend more time with their families, more flexible work hours should be encouraged [40]. Connor & Davidson defined resilience as a measure of stress-coping ability [25]. Optimism, perseverance, self-efficacy, flexibility, and emotional awareness are factors contributing to a person’s resilience [16, 17]. The results of our study showed that participants who recorded high grit scores and more resilience were less likely to experience EE and DP. Interestingly, resilience is teachable; the U.S. army included resilience training in their programs [46, 47]. We suggest that seminars about girt and resilience training may be helpful in reducing the prevalence of burnout among orthopedic surgeons. In addition, taking into consideration the higher prevalence of burnout among residents, it is crucial to develop and provide adequate support systems for them and establish national guidelines for maximum weekly working hours. Furthermore, individual-level actions to reduce stress through healthy lifestyles and behaviors and by promoting effective coping strategies might be helpful [48].

The strength of our study is that it addresses the relationship between grit and resilience to burnout using various validated scales among orthopedic surgeons in Jordan. In addition, to the best of our knowledge, no previous study reported the prevalence and factors associated with burnout among orthopedic surgeons in Jordan. Moreover, our study had a high response rate of 75% compared to the low response rates reported in similar studies. However, the study has some limitations including but not limited to: the cross-sectional design, non-random sampling, and humble sample size. Further prospective studies with a larger sample size using a randomized sampling method are needed to better examine associations.

## Conclusion

The practice of orthopedic surgery in Jordan is associated with a high rate of burnout, especially among resident surgeons. Several risk factors, including weekly working hours, were documented, and grit and resilience may have a protective effect. We suggest that healthcare professionals, including orthopedic care providers, be involved in programs focusing on coping with stress and increasing self-esteem, grit, and resilience.

## Data Availability

The dataset used during the current study is available from the corresponding author upon reasonable request.
